# Association between triglyceride glucose-body mass index and all-cause mortality in critically ill patients with acute myocardial infarction: retrospective analysis of the MIMIC-IV database

**DOI:** 10.3389/fnut.2024.1399969

**Published:** 2024-06-18

**Authors:** Chaodi Luo, Qian Li, Zhuoer Wang, Sifan Duan, Qiang Ma

**Affiliations:** ^1^Department of Peripheral Vascular Diseases, First Affiliated Hospital of Xi’an Jiaotong University, Xi’an, China; ^2^Department of Cardiology, First Affiliated Hospital of Xi’an Jiaotong University, Xi’an, China; ^3^Medical College of Xi’an Jiaotong University, Xi’an, China

**Keywords:** triglyceride-glucose-body mass index, acute myocardial infarction, insulin resistance, prognosis, all-cause mortality

## Abstract

**Background:**

Insulin resistance (IR) is closely related to the development of cardiovascular diseases. Triglyceride-glucose-body mass index (TyG-BMI) has been proven to be a reliable surrogate of IR, but the relationship between TyG-BMI and acute myocardial infarction (AMI) is unknown. The present study aims to determine the effects of TyG-BMI on the clinical prognosis of critically ill patients with AMI.

**Methods:**

The data of AMI patients were extracted from the Medical Information Mart for Intensive Care IV (MIMIC-IV) database. All patients were divided into four groups according to the TyG-BMI quartile. Outcomes were defined as 30-, 90-, 180-, and 365-day all-cause mortality. Kaplan–Meier (K-M) curve was used to compare survival rate between groups. Meanwhile, Cox regression analysis and restricted cubic splines (RCS) were used to explore the relationship between TyG-BMI index and outcome events.

**Results:**

A total of 1,188 critically ill patients with AMI were included in this study. They were divided into four groups according to TyG-BMI quartiles, there were significant differences in 90-, 180-, and 365-day all-cause mortality while there was no difference in 30-day all-cause mortality. Interestingly, with the increase of TyG-BMI, the 90-, 180-, and 365-day survival rate increased first and then gradually decreased, but the survival rate after decreasing was still higher than that in the group with the lowest TyG-BMI. U-shaped relationships between TyG-BMI index and 90-, 180-, and 365-day all-cause mortality were identified using RCS curve and the inflection point was 311.1, 316.5, and 320.1, respectively, whereas the TyG-BMI index was not non-linearly associated with 30-day all-cause mortality. The results of Cox proportional hazard regression analysis are consistent with those of RCS analysis.

**Conclusion:**

U-shaped relationships are existed between the TyG-BMI index and 90-, 180-, and 365-day all-cause mortality in critically ill patients with AMI, but not 30-day all-cause mortality. The TyG-BMI index can be used as an effective index for early prevention of critically ill patients with AMI.

## Introduction

Over past decades, cardiovascular disease has long been one of the leading causes of death worldwide. As a common cardiovascular disease, acute myocardial infarction (AMI) is always complicated by heart failure (HF), malignant ventricular arrhythmia, and cardiogenic shock, which increase economic and social burden on countries around the world (1–3). Although revascularization and pharmacological strategies for patients with AMI have been optimized, the incidence of major adverse cardiovascular events remains high, especially in critically ill patients (4). However, there are still lacking studies which could simply demonstrate the prognosis of critically ill patients with AMI. Therefore, it is essential to clarify and avoid risk factors to reduce complications and mortality in critically ill patients with AMI.

Insulin resistance (IR) is a decrease in the sensitivity of insulin target cells or tissues to insulin, and a reduction in the efficiency of insulin in promoting glucose uptake, which results in these cells or tissues require large amounts of insulin to produce normal biological effects (5). Previous studies have suggested that IR is considered to be one of the most important causes of cardiovascular disease, type 2 diabetes, non-alcoholic fatty liver disease (NAFLD), and polycystic ovary syndrome (6–8). Yang et al. (9) studied 485 non-diabetic patients with ST elevated myocardial infarction (STEMI) and showed that the state of IR and glycemic abnormalities were prevalent in patients with STEMI, which is a significant predictor of left ventricular dilatation after AMI. There is a need to pay more attention to patients with risk factors for mortality at admission, since the mortality rate for AMI patients with IR is higher than for non-IR AMI patients (10).

In recent years, the triglyceride-glucose (TyG) index was proposed to be a simple surrogate marker of IR, and it has been proved to predict the prognosis of cardiovascular disease, including coronary heart disease (CHD), HF, AMI, stroke, and hypertension (11–13). Base on TyG index, the triglyceride-body mass index (TyG-BMI) was proposed. The TyG-BMI index added BMI to the TyG index, which significantly improves its predictive performance. Er et al. (14) conducted a study and investigated the efficiency of several combinations of the TyG and obesity indices including BMI, waist circumference (WC), and waist-to-height ratio (WHtR) in reflecting IR, indicating that TyG-BMI index had the most significant association with HOMA-IR compared to other markers and believed it had more diagnostic significance than the TyG index. Meanwhile, previous studies mainly reported that TyG-BMI was associated with hypertension, prediabetes, hyperuricemia, and NAFLD (15–18). There is still uncertainty regarding the association between TyG-BMI index and AMI, especially in critically ill patients with AMI. Therefore, the present study aimed to investigate the relationship between the TyG-BMI index and all-cause mortality in critically ill patients with AMI.

## Methods

### Data source

The data analyzed in this study were all obtained from the Medical Information Mart for Intensive Care IV (MIMIC IV, version 2.2) database, which is a large, publicly accessible database that is developed and managed by the computational physiology laboratory of Massachusetts Institute of Technology (MIT). Over 190,000 patients have been admitted to Beth Israel Deaconess Medical Center (BIDMC) between 2008 and 2019. Data were collected on each patient’s demographic information, laboratory tests, medication treatment, vital signs, and follow-up information. The laboratory data obtained were the first measurements taken when the patients were first admitted to the ICU, and were not affected by factors such as fasting and parenteral nutrition. All patients’ information was de-identified by replacing patient identification with random codes, so informed consent or ethical approval did not need to be obtained from patients.

### Data extraction

The data analyzed in the study were all extracted by using structure query language (SQL) in Navicat Premium (Version 16.1.15). All required data of patients were extracted, including demographics [age, gender, body mass index (BMI), and smoking]; vital signs [systolic blood pressure (SBP), diastolic blood pressure (DBP), mean blood pressure (MBP), heart rate (HR), and respiratory rate (RR)]; medications [aspirin, clopidogrel, statin, beta-blockers, digitalis, angiotensin-converting enzyme inhibitor (ACEI) and angiotensin receptor blocker (ARB), calcium channel blockers (CCB), diuretics, norepinephrine, phenylephrine, vasopressin, epinephrine, dopamine, dobutamine, and insulin use]; comorbidities [atrial fibrillation (AF), hypertension, diabetes, chronic obstructive pulmonary disease (COPD), congestive heart failure (CHF), peripheral vascular disease (PVD), cerebrovascular disease (CVD), dementia, and rheumatic disease]; procedures [percutaneous coronary intervention, coronary artery bypass grafting (CABG)]; laboratory markers [pH, SpO_2_, SO_2_, PaO_2_, PaCO_2_, bicarbonate, base excess (BE), anion gap, lactate, albumin, urea nitrogen (BUN), creatinine, fasting glucose, calcium, chloride, sodium, potassium, prothrombin time (PT), partial thromboplastin time (PTT), hematocrit, hemoglobin, platelets, white blood cell (WBC), lymphocytes, neutrophils, mean corpuscular hemoglobin (MCH), mean corpuscular hemoglobin concentration (MCHC), mean corpuscular volume, red blood cell (RBC), red blood cell distribution width (RDW), high-density lipoprotein cholesterol (HDL-C), low-density lipoprotein cholesterol (LDL-C), triglycerides (TG), total cholesterol (TC), HbA1c, Glasgow Coma Scale (GCS) sore, Sepsis-related Organ Failure (SOFA), and Simplified Acute Physiology Score II (SAPSII)]; endpoint events [cardiac arrest, cardiogenic shock, length of stay (LOS) in hospital, LOS in ICU, ICU mortality, 30-day mortality, 90-day mortality, 180-day mortality, and 365-day mortality].

### Population selection criteria

**Figure 1:**
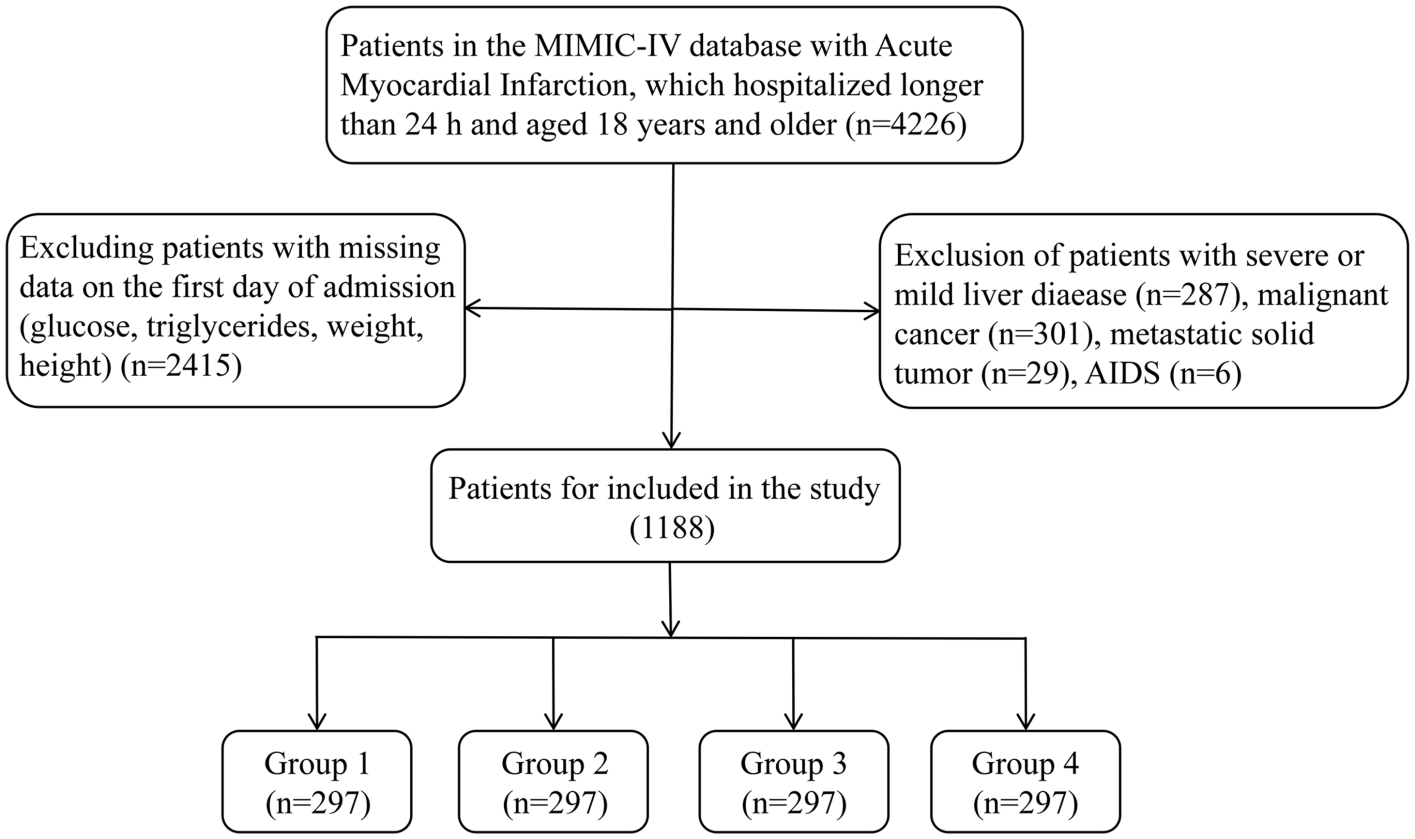
Flowchart of the included patients.

AMI patients who were admitted for the first time and transferred into intensive care unit (ICU) were included in our study. All patients with AMI were diagnosed and identified according to International Classification of Diseases, Ninth Revision (ICD-9) and Tenth Revision (ICD-10). The ICD-9 and ICD-10 code were 410% and I21%, respectively. These patients would be excluded after further screening: (1) patients stayed in ICU less than 24 h; (2) patients without recorded data including fasting glucose, triglycerides, weight, and height at admission; (3) patients admitted with acquired immune deficiency syndrome (AIDS), metastatic solid tumor, sever or mild liver disease, and malignant cancer. Ultimately, 1,188 patients were enrolled in the study (Figure 1).

### TyG-BMI index calculation formula

The TyG index was calculated as ln [fasting glucose (mg/dL) × fasting TG (mg/dL)]/2 (12). Meanwhile, BMI was calculated as body weight (Kg)/height^2^ (m). The TyG-BMI index was determined based on the combination of TyG index and BMI. TyG-BMI index was calculated according the equation: TyG index × BMI (14).

### Grouping and endpoint events

Patients were categorized into four groups in this study based on quartiles of TyG-BMI levels: Q 1 (*n* = 297, TyG-BMI < 240.6), Q 2 (*n* = 297, 240.6 ≤ TyG-BMI < 278.8), Q 3 (*n* = 297, 278.8 ≤ TyG-BMI <329.5), and Q 4 (*n* = 297, TyG-BMI ≥ 329.5). The study endpoint events were 30-, 90-, 180-, and 365-day all-cause mortality.

### Statistical analysis

The Kolmogorov–Smirnov test was used to assess the normality of continuous variables. Data were presented as mean and standard deviation (SD) for normally distributed continuous variables, median and interquartile range (IQR) for non-normal distributed continuous variables, and as a number and percentages for categorical variables. Continuous variables were compared by using Student’s *t*-test or one-way ANOVA and categorical variables were examined by using Fisher’s exact test or Chi-squared test.

To explore the relationship between TyG-BMI index and clinical outcomes, Cox regression analysis models were performed to explore the association between TyG-BMI index (as categorical metrics in quartiles) and endpoints (30-, 90-, 180-, and 365-day all-cause mortality). Model 1 was only included TyG-BMI index, model 2 adjusted for variables in model 1 plus age, gender, SBP, hypertension, diabetes, AF, CHF, and length of stay in ICU, model 3 adjusted for variables in model 2 plus aspirin, clopidogrel, beta blockers, insulin, statin, model 4 adjusted for variables in model 3 plus HDL-C, WBC, BUN, creatinine. In the present study, the variables whose *p* < 0.05 in univariate regression analysis into the multivariate regression analysis. Meanwhile, the variables known to be significantly associated with the prognosis of AMI were also included in the multivariate regression analysis model, even if they did not meet established statistical screening criteria. Then the moderating variables were, respectively, included in models 2–4 for analysis. Kaplan–Meier (K-M) survival curves were adopted to investigate the incidence of endpoints by stratifying by TyG-BMI index, and the log-rank test was used. The TyG-BMI index was also analyzed as a continuous variable using restricted cubic splines (RCS) to clarify the dose-effect correlations with the risk of major and secondary endpoint events. In addition, subgroup analyses were conducted to examine the effects of TyG-BMI in different subgroups, such as sex, age, hypertension, and diabetes. All statistical analyses were performed in R version 4.3.2 (R Foundation for Statistical Computing, Vienna, Austria) using R Studio version 2023.09.1 + 494. *p* values were obtained using a two-tailed test, and *p* < 0.05 was considered to indicate a statistically significant difference.

## Results

### Baseline characteristics

1,188 critically ill patients with AMI were finally included in the study, among which were divided into four groups based on TyG-BMI quartiles. The patients included in the study had an average age of 69.1 ± 13.1 years and the percentage of male was 64.7%. Baseline clinical characteristics of patients stratified by TyG-BMI were analyzed and shown in Table 1. The levels of TyG-BMI index of the four groups were 211.3 ± 21.7, 260.8 ± 10.6, 302.4 ± 14.5, and 387.7 ± 56.0, respectively. Patients with the highest quartile group were the youngest with an average age of 65.2. It was clear to see that BMI, PaCO_2_, hemoglobin, WBC, RBC, BUN, creatinine, potassium, TG, TC, and HbA1c of patients increased with the increase of TyG-BMI index, while SaO_2_, PH, chloride, sodium, and HDL-C gradually decreased with the increase of TyG-BMI index. Interestingly, we found that the incidence of AF was lower in group Q4 than in other groups and the incidence of hypertension had no difference between those groups. Meanwhile, 90-, 180-, and 365-day all-cause mortality did not decrease with increasing TyG-BMI index, which was lowest in group Q3 and then increased in group Q4.

**Table 1:** Baseline characteristics of patients grouped according to TyG-BMI index quartiles.

Variables	Q 1 (*N* = 297)	Q 2 (*N* = 297)	Q 3 (*N* = 297)	Q 4 (*N* = 297)	p
Age, years	73.0 ± 14.0	71.4 ± 12.4^*^	67.6 ± 12.8^*^	65.2 ± 11.7	<0.001
Male, *n* (%)	183 (61.6)	210 (70.7)	207 (69.7)	169 (56.9)	<0.001
Weight, Kg	63.8 ± 10.9	77.5 ± 10.8^***^	87.4 ± 12.3^***^	106.5 ± 19.9^***^	<0.001
Height, cm	168.7 ± 10.1	170.5 ± 9.8^*^	170.7 ± 10.2^*^	169.1 ± 11.1	0.044
BMI, Kg/m^2^	22.5 ± 2.5	26.9 ± 2.0^***^	30.3 ± 2.3^***^	37.6 ± 5.72^***^	<0.001
Smoking, *n* (%)	31 (10.4)	17 (5.7)^*^	28 (9.4)	24 (8.1)	0.187
Past history, *n* (%)					
AF	116 (39.1)	110 (37)	105 (35.4)	79 (26.6)^**^	0.008
Hypertension	221 (74.4)	226 (76.1)	236 (79.5)	246 (82.8)^*^	0.063
Diabetes	81 (27.3)	106 (35.7)	129 (43.4)^***^	174 (58.6)^***^	<0.001
COPD	90 (30.3)	64 (21.5)^*^	66 (22.2)^*^	76 (25.6)	0.054
CHF	151 (50.8)	146 (49.2)	151 (50.8)	171 (57.6)	0.174
PVD	57 (19.2)	45 (15.2)	53 (17.8)	46 (15.5)	0.500
CVD	45 (15.2)	49 (16.5)	44 (14.8)	49 (16.5)	0.913
Dementia	13 (4.4)	5 (1.7)	6 (2.0)	6 (2.0)	0.132
Rheumatic disease	11 (3.7)	14 (4.7)	12 (4.0)	8 (2.7)	0.630
Vital signs					
SBP, mmHg	122.4 ± 21.8	121.8 ± 23.7	121.4 ± 21.3	123.2 ± 25.0	0.800
DBP, mmHg	65.1 ± 16.8	65.8 ± 17.4	68.6 ± 16.9	67.3 ± 19.2	0.076
MBP, mmHg	82.7 ± 16.1	83.3 ± 16.2	85.1 ± 19.1	83.3 ± 19.7	0.372
HR, beats/min	85.3 ± 17.5	83.5 ± 18.0	86.3 ± 19.0	87.3 ± 17.4	0.060
RR, times/min	18.2 ± 5.8	17.7 ± 5.0	18.7 ± 5.7	18.8 ± 5.4	0.058
Medication, *n* (%)					
Aspirin	278 (93.6)	280 (94.3%)	289 (97.3%)	278 (93.6%)	0.136
Clopidogrel	139 (46.8)	133 (44.8)	140 (47.1)	135 (45.5)	0.931
Beta blockers	259 (87.2)	268 (90.2)	275 (92.6)	249 (83.8)	0.006
ACEI/ARB	48 (16.2)	45 (15.2)	57 (19.2)	50 (16.8)	0.599
CCB	13 (4.4)	15 (5.1)	19 (6.4)	21 (7.1)	0.476
Digitalis	20 (6.7)	13 (4.4)	14 (4.7)	14 (4.7)	0.547
Diuretics	221 (74.4)	224 (75.4)	205 (69)	233 (78.5)	0.065
Norepinephrine	68 (22.9)	62 (20.9)	71 (23.9)	88 (29.6)	0.078
Phenylephrine	77 (25.9)	81 (27.3)	69 (23.2)	67 (22.6)	0.500
Vasopressin	13 (4.4)	22 (7.4)	24 (8.1)	28 (9.4)	0.112
Epinephrine	21 (7.1)	35 (11.8)	32 (10.8)	38 (12.8)	0.119
Dopamine	23 (7.7)	19 (6.4)	18 (6.1)	20 (6.7)	0.861
Dobutamine	5 (1.7)	10 (3.4)	13 (4.4)	15 (5.1)	0.140
Insulin	167 (56.2)	184 (62)	184 (62)	218 (73.4)^***^	<0.001
Statin	256 (86.2)	257 (86.5)	265 (89.2)	262 (88.2)	0.644
Laboratory data					
SpO_2_, %	99.0 (96.0–100.0)	99.0 (96.0–100.0)	98.0 (95.0–100.00)	98.0 (95.0–100.0)	0.013
SO_2_, Median (IQR)	96.0 (91.0–98.0)	97.0 (94.0–98.0)	97.0 (92.0–98.00)	96.0 (89.0–97.0)	0.008
PO_2_, Median (IQR)	133.0 (62.0–370.0)	172.5 (69.5–390.0)	142.5 (69.0–337.00)	130.0 (66.5–291.0)	0.132
PaCO_2_, Median (IQR)	40.0 (34.0–46.0)	40.0 (35.0–46.0)	40.5 (36.0–45.50)	43.0 (37.0–50.0)	0.005
Lactate, Median (IQR)	1.50 (1.10–2.50)	1.50 (1.10–2.30)	1.50 (1.20–2.50)	1.60 (1.20–2.80)	0.303
pH, Mean ± SD	7.36 ± 0.11	7.36 ± 0.11	7.35 ± 0.10	7.32 ± 0.12^**^	0.003
BE, Median (IQR)	−0.50 (−5.00 to 1.00)	0.00 (−4.00 to 1.00)	0.00 (−5.50 to 1.00)	−1.00 (−5.00 to 1.00)	0.311
Albumin, Mean ± SD	3.42 ± 0.66	3.41 ± 0.63	3.55 ± 0.57	3.36 ± 0.66	0.144
Anion gap, Mean ± SD	15.8 ± 5.3	15.7 ± 5.0	15.9 ± 4.6	16.7 ± 5.2^*^	0.059
Bicarbonate, Mean ± SD	22.8 ± 4.7	22.3 ± 4.4	22.4 ± 4.2	22.2 ± 4.9	0.898
Glucose, Median (IQR)	127.0 (107.0–161.0)	136.0 (108.0–184.0)	140.0 (111.0–192.0)^**^	168.0 (130.0–248.0)^***^	<0.001
BUN, Median (IQR)	21.0 (16.0–33.0)	21.0 (15.0–29.0)	20.0 (15.0–30.0)	22.0 (16.0–35.0)	0.036
Calcium, Mean ± SD	8.52 ± 0.75	8.61 ± 0.79	8.65 ± 0.80^*^	8.57 ± 0.81	0.228
Chloride, Mean ± SD	104.0 ± 7.3	103.2 ± 5.6	103.1 ± 6.4	102.0 ± 6.0^***^	0.002
Creatinine, Median (IQR)	1.10 (0.80–1.50)	1.10 (0.90–1.60)	1.10 (0.90–1.50)	1.20 (0.90–1.90)^*^	0.004
Sodium, Mean ± SD	138.8 ± 6.0	138.1 ± 4.1	137.9 ± 4.5^*^	137.7 ± 4.6^**^	0.037
Potassium, Mean ± SD	4.33 ± 0.74	4.34 ± 0.75	4.35 ± 0.79	4.50 ± 0.81^**^	0.027
PT, Median (IQR)	13.4 (12.2–15.7)	13.4 (12.0–15.4)	13.4 (12.1–15.5)	13.6 (12.1–15.5)	0.859
PTT, Median (IQR)	32.2 (27.6–44.6)	34.0 (27.6–61.9)	32.6 (27.8–52.6)	31.2 (27.2–52.1)	0.254
Hematocrit, Mean ± SD	34.2 ± 7.5	34.5 ± 7.3	35.6 ± 7.4^*^	35.5 ± 7.4^*^	0.035
Hemoglobin, Mean ± SD	11.2 ± 2.5	11.5 ± 2.5	11.9 ± 2.6^**^	11.7 ± 2.5^*^	0.014
Platelets, Mean ± SD	228.7 ± 116.0	220.8 ± 106.0	225.0 ± 86.3	232.7 ± 93.5	0.519
WBC, Mean ± SD	12.3 ± 6.2	12.0 ± 5.8	13.2 ± 6.2	14.3 ± 6.6^***^	<0.001
Lymphocytes, Mean ± SD	1.39 ± 0.78	1.66 ± 0.93^**^	1.83 ± 1.13^***^	1.68 ± 1.07^**^	<0.001
Neutrophils, Mean ± SD	10.3 ± 6.1	10.7 ± 5.8	11.3 ± 6.3	12.2 ± 5.9^**^	0.012
MCH, Mean ± SD	30.4 ± 2.6	30.3 ± 2.2	30.0 ± 2.3^**^	29.5 ± 2.3^***^	<0.001
MCHC, Mean ± SD	33.0 ± 1.7	33.3 ± 1.6^**^	33.3 ± 1.6^**^	32.8 ± 1.6	<0.001
MCV, Mean ± SD	92.3 ± 6.78	91.2 ± 6.4^*^	90.2 ± 5.8^***^	90.0 ± 6.4^***^	<0.001
RBC, Mean ± SD	3.71 ± 0.86	3.81 ± 0.84	3.96 ± 0.86^***^	3.97 ± 0.88^***^	<0.001
RDW, Mean ± SD	14.4 ± 2.0	14.2 ± 2.0	14.3 ± 1.6	14.5 ± 1.9	0.390
HDL-C, Mean ± SD	50.6 ± 17.2	48.3 ± 19.3	43.4 ± 13.3^***^	42.5 ± 15.3^***^	<0.001
LDL-C, Mean ± SD	85.8 ± 45.4	88.9 ± 39.1	93.56 ± 41.2	92.9 ± 40.2	0.115
TG, Mean ± SD	101.0 ± 49.0	127.2 ± 73.1^**^	167.3 ± 117.7^***^	211.8 ± 179.6^***^	<0.001
TC, Mean ± SD	157.0 ± 53.5	162.1 ± 51.2	167.7 ± 49.7^**^	173.1 ± 53.4^***^	0.003
HbA1c, Mean ± SD	6.21 ± 1.28	6.44 ± 1.47	6.58 ± 1.71^*^	7.21 ± 1.85^***^	<0.001
TyG-BMI	211.3 ± 21.7	260.8 ± 10.6^***^	302.4 ± 14.5^***^	387.7 ± 56.0^***^	<0.001
GCS, Median (IQR)	14.0 (11.0–15.0)	14.0 (12.0–15.0)	14.0 (10.0–15.0)	14.0 (10.0–15.0)	0.990
SAPSII, Median (IQR)	37.0 (29.0–46.0)	36.0 (29.0–46.0)	34.0 (26.0–47.0)	37.0 (28.0–47.0)	0.384
SOFA, Median (IQR)	5.0 (2.0–8.0)	5.0 (3.0–9.0)	5.0 (2.0–9.0)	6.0 (3.0–10.0)^*^	0.046
CABG, *n* (%)	82 (27.6)	111 (37.4)	96 (32.3)	94 (31.6)	0.088
PCI, *n* (%)	58 (19.5)	53 (17.8)	66 (22.2)	49 (16.5)	0.318
Outcome					
Cardiac arrest, *n* (%)	15 (5.1)	22 (7.4)	18 (6.1)	26 (8.8)	0.303
Cardiogenic shock, *n* (%)	50 (16.8)	51 (17.2)	53 (17.8)	52 (17.5)	0.990
LOS in hospital, Median (IQR)	8.9 (5.9–14.0)	8.8 (5.0–13.9)	8.9 (5.0–14.2)	9.8 (5.9–14.7)	0.330
LOS in ICU, Median (IQR)	3.1 (1.8–5.3)	2.9 (1.6–5.9)	3.1 (1.7–6.2)	3.0 (1.8–6.5)	0.781
ICU mortality, *n* (%)	20 (6.7)	24 (8.1)	19 (6.4)	31 (10.4)	0.250
30-day mortality, *n* (%)	39 (13.1)	36 (12.1)	33 (11.1)	38 (12.8)	0.884
90-day mortality, *n* (%)	64 (21.5)	50 (16.8)	34 (11.4)^**^	48 (16.2)	0.011
180-day mortality, *n* (%)	79 (26.6)	62 (20.9)	40 (13.5)^***^	52 (17.5)^*^	<0.001
365-day mortality, *n* (%)	95 (32)	74 (24.9)^*^	53 (17.8)^***^	63 (21.2)^**^	<0.001

Continuous variables are presented as mean ± SD if normally distributed, and median (interquartile range) if not normally distributed. Categorical variables are presented as number of patients (%). ^*^Compared with Q1 group, *p* < 0.05, ^**^Compared with Q1 group, *p* < 0.01, ^***^Compared with Q1 group, *p* < 0.001. BMI, Body mass index; AF, Atrial fibrillation; COPD, Chronic obstructive pulmonary disease; CHF, Congestive heart failure; PVD, Peripheral vascular disease; CVD, Cerebrovascular disease; SBP, Systolic blood pressure; DBP, Diastolic blood pressure; MBP, Mean blood pressure; HR, Heart rate; RR, Respiratory rate; PaCO_2_, Carbon dioxide partial pressure; PaO_2_, Partial pressure of arterial oxygen; BE, Base excess; BUN, Urea nitrogen; PT, Prothrombin time; PTT, Partial prothrombin time; WBC, White blood cell; MCH, Mean corpuscular hemoglobin; MCHC, Mean corpusular hemoglobin concerntration; MCV, Mean corpuscular volume; RBC, Red blood cell; RDW, Red cell distribution width; HDL-C, High density lipoprotein cholesterol; LDL-C, Low density lipoprotein cholesterol; TC, Total cholesterol; TG, Triglyceride; GCS, Glasgow coma scale; SOFA, Sequential organ failure assessment; SAPS II, Simplified acute physiology score; PCI, Percutaneous coronary intervention; and CABG, Coronary artery bypass grafting.

### Study endpoints

**Figure 2:**
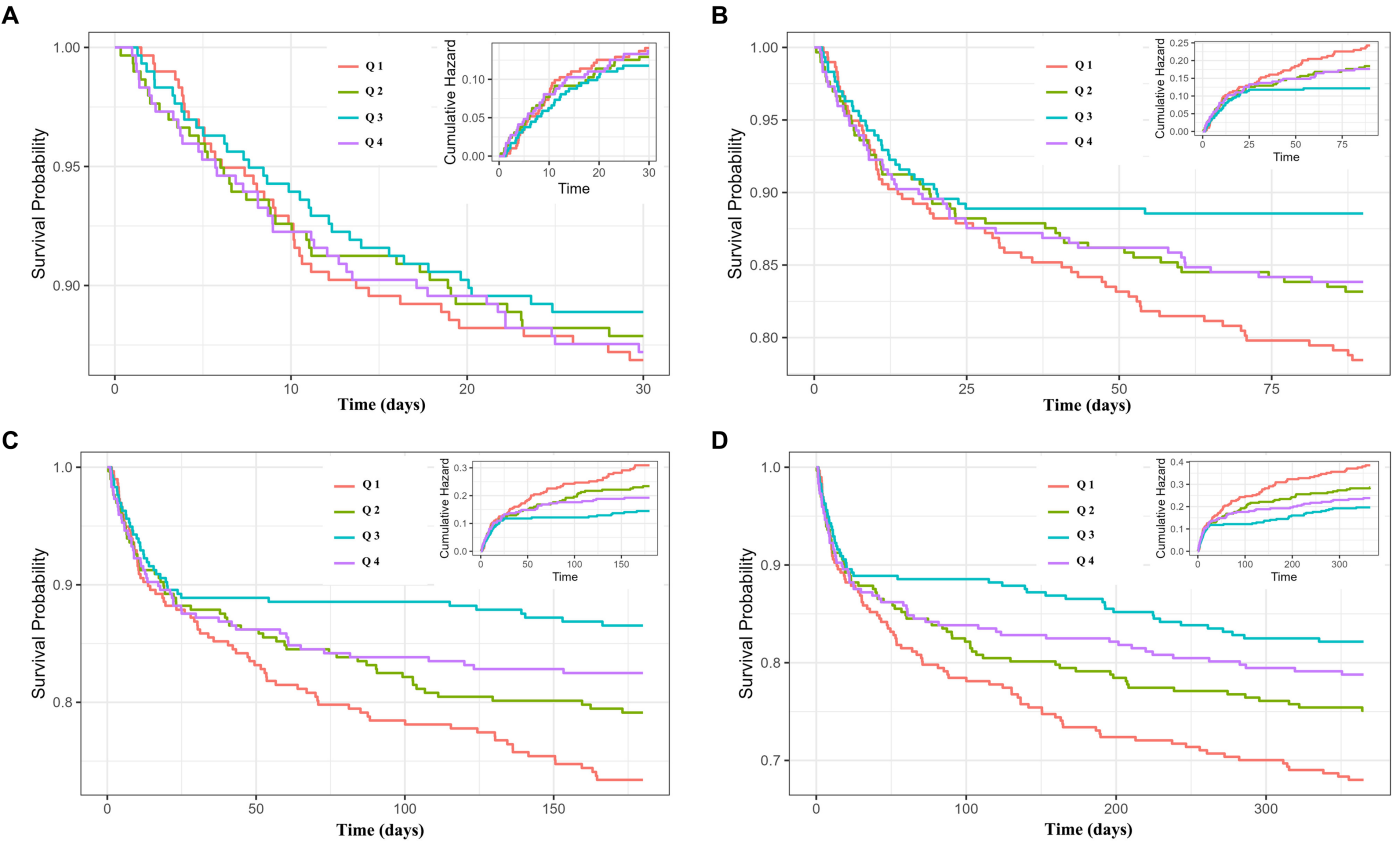
Cumulative incidence and Kaplan–Meier survival analysis curves for all-cause mortality. TyG-BMI index: Q 1 (TyG-BMI < 240.6), Q 2 (240.6 ≤ TyG-BMI < 278.8), Q 3 (278.8 ≤ TyG-BMI <329.5), and Q 4 (TyG-BMI ≥ 329.5). Cumulative incidence and Kaplan–Meier curves showing the cumulative probability of all-cause mortality according to groups at 30-day **(A)**, 90-day **(B)**, 180-day **(C)**, and 365-day **(D)**.

Further investigation of the incidence of all-cause mortality in AMI patients based on the TyG-BMI quartiles and the relationship between the TyG-BMI and all-cause mortality would be undertaken. The 30-day survival rates of the four groups were not significantly different based on the K-M curve analysis (Figure 2A). However, the influence of TyG-BMI on the 365-day all-cause mortality of patients was different. With the increase of TyG-BMI, the 365-day survival rate increased first and then gradually decreased, but the survival rate after decreasing was still higher than that in the group with the lowest TyG-BMI (Figure 2D). Meanwhile, K-M curve analysis also indicated the trends of the effect of TyG-BMI among the four groups on 90- and 180-day all-cause mortality were consistent with that on 365-day all-cause mortality (Figures 2B,C).

### The association between TyG-BMI and the all-cause mortality of AMI

The relationship between TyG-BMI index and all-cause mortality was examined using Cox proportional hazard regression model when TyG-BMI index was used as a continuous variable. As shown in Table 2, TyG-BMI index had no association with 30-day all-cause mortality, even after adjusting for confounding factors. In addition, four models were also examined the association between TyG-BMI index and 90-, 180-, and 365-day all-cause mortality. After adjustment for age, gender, SBP, hypertension, diabetes, AF, CHF, length of stay in ICU, aspirin, clopidogrel, beta blockers, insulin, statin, HDL-C, WBC, BUN, and creatinine, the hazard ratios (HR) and 95% confidence intervals (CIs) from lowest to highest TyG-BMI index categories were 1.000 (reference), 0.909 (0.587–1.409), 0.570 (0.341–0.952), and 0.732 (0.452–1.187), respectively, for 90-day all-cause mortality (Table 3); 1.000 (reference), 0.899 (0.609–1.328), 0.489 (0.306–0.782), and 0.639 (0.414–0.986), respectively, for 180-day all-cause mortality (Table 4); 1.000 (reference), 0.863 (0.607–1.227), 0.514 (0.339–0.781), and 0.658 (0.448–0.967), respectively, for 365-day all-cause mortality (Table 5). The results of Cox proportional hazard regression analysis are consistent with those of K-M curve analysis.

**Table 2:** Cox proportional hazard models for 30-day all-cause mortality.

Variables	Q 1 (Ref.)	Q 2 HR (95% CI)	Q 3 HR (95% CI)	Q 4 HR (95% CI)
Model 1^a^	1.000	0.924 (0.587–1.454)	0.839 (0.527–1.333)	0.977 (0.625–1.528)
*p* value	-	0.733	0.457	0.920
Model 2^b^	1.000	0.988 (0.622–1.572)	1.018 (0.628–1.648)	1.190 (0.730–1.941)
*p* value	-	0.961	0.943	0.486
Model 3^c^	1.000	1.069 (0.672–1.702)	1.046 (0.636–1.722)	1.309 (0.803–2.134)
*p* value	-	0.777	0.858	0.280
Model 4^d^	1.000	1.059 (0.622–1.803)	0.709 (0.391–1.287)	1.128 (0.659–1.931)
*p* value	-	0.832	0.259	0.661

^a^Model 1: Univariate model.

^b^Model 2: Adjusted for Age, gender, SBP, hypertension, diabetes, atrial fibrillation, congestive heart failure, and length of stay in ICU.

^c^Model 3: Adjusted for model 2 plus aspirin, clopidogrel, beta blockers, insulin, and statin.

^d^Model 4: Adjusted for model 4 plus HDL-C, WBC, BUN, and creatinine.

**Table 3:** Cox proportional hazard models for 90-day all-cause mortality.

Variables	Q 1 (Ref.)	Q 2 HR (95% CI)	Q 3 HR (95% CI)	Q 4 HR (95% CI)
Model 1^a^	1.000	0.775 (0.535–1.121)	0.518 (0.342–0.785)	0.745 (0.512–1.082)
*p* value	-	0.176	0.002	0.122
Model 2^b^	1.000	0.812 (0.555–1.190)	0.637 (0.414–0.980)	0.917 (0.607–1.384)
*p* value	-	0.286	0.040	0.679
Model 3^c^	1.000	0.872 (0.595–1.276)	0.777 (0.503–1.200)	0.826 (0.544–1.254)
*p* value	-	0.480	0.255	0.369
Model 4^d^	1.000	0.909 (0.587–1.409)	0.570 (0.341–0.952)	0.732 (0.452–1.187)
*p* value	-	0.671	0.032	0.206

^a^Model 1: Univariate model.

^b^Model 2: Adjusted for age, gender, SBP, hypertension, diabetes, atrial fibrillation, congestive heart failure, and length of stay in ICU.

^c^Model 3: Adjusted for model 2 plus aspirin, clopidogrel, beta blockers, insulin, and statin.

^d^Model 4: Adjusted for model 4 plus HDL-C, WBC, BUN, and Creatinine.

**Table 4:** Cox proportional hazard models for 180-day all-cause mortality.

Variables	Q 1 (Ref.)	Q 2 HR (95% CI)	Q 3 HR (95% CI)	Q 4 HR (95% CI)
Model 1^a^	1.000	0.773 (0.554–1.078)	0.485 (0.331–0.709)	0.646 (0.455–0.916)
*p* value	-	0.129	<0.001	0.014
Model 2^b^	1.000	0.771 (0.547–1.088)	0.582 (0.393–0.861)	0.781 (0.533–1.144)
*p* value	-	0.139	0.007	0.204
Model 3^c^	1.000	0.857 (0.608–1.207)	0.668 (0.449–0.993)	0.689 (0.468–1.014)
*p* value	-	0.377	0.046	0.059
Model 4^d^	1.000	0.899 (0.609–1.328)	0.489 (0.306–0.782)	0.639 (0.414–0.986)
*p* value	-	0.592	0.003	0.043

^a^Model 1 Univariate model.

^b^Model 2: Adjusted for age, gender, SBP, hypertension, diabetes, atrial fibrillation, congestive heart failure, and length of stay in ICU.

^c^Model 3: Adjusted for model 2 plus aspirin, clopidogrel, beta blockers, insulin, and statin.

^d^Model 4 adjusted for model 4 plus HDL-C, WBC, BUN, and creatinine.

**Table 5:** Cox proportional hazard models for 365-day all-cause mortality.

Variables	Q 1 (Ref.)	Q 2 HR (95% CI)	Q 3 HR (95% CI)	Q 4 HR (95% CI)
Model 1^a^	1.000	0.758 (0.559–1.027)	0.522 (0.373–0.731)	0.638 (0.464–0.877)
*p* value	-	0.074	<0.001	0.006
Model 2^b^	1.000	0.789 (0.577–1.080)	0.619 (0.437–0.876)	0.742 (0.524–1.051)
*p* value	-	0.139	0.007	0.093
Model 3^c^	1.000	0.839 (0.614–1.148)	0.680 (0.478–0.966)	0.721 (0.507–1.024)
*p* value	-	0.273	0.031	0.068
Model 4^d^	1.000	0.863 (0.607–1.227)	0.514 (0.339–0.781)	0.658 (0.448–0.967)
*p* value	-	0.412	0.002	0.033

^a^Model 1: Univariate model.

^b^Model 2: Adjusted for age, gender, sbp, hypertension, diabetes, atrial fibrillation, congestive heart failure, and length of stay in ICU.

^c^Model 3: Adjusted for model 2 plus aspirin, clopidogrel, beta blockers, insulin, and statin.

^d^Model 4 adjusted for model 4 plus HDL-C, WBC, BUN, and creatinine.

### Analysis of nonlinear relationships

**Figure 3:**
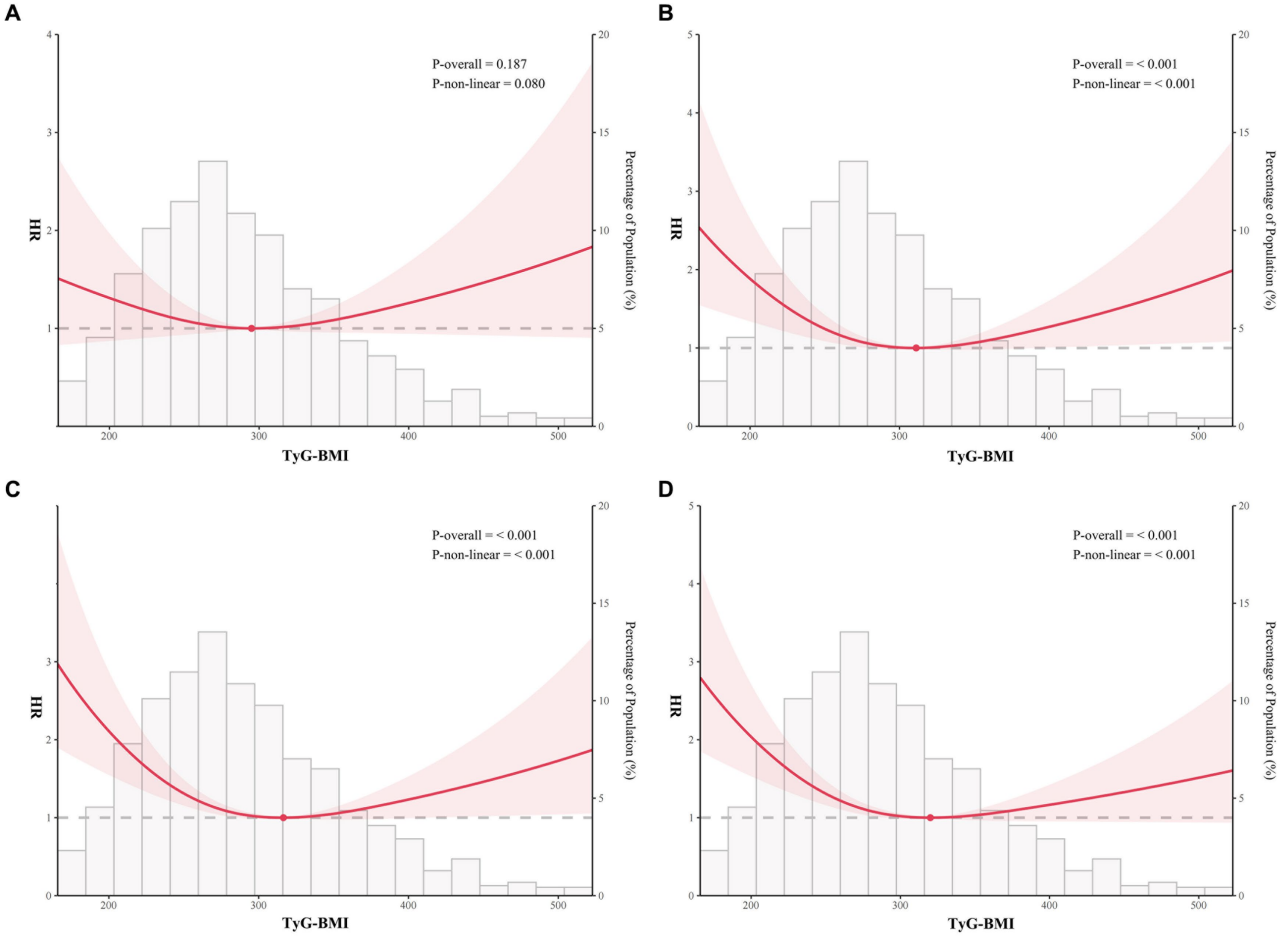
Restricted cubic spline analysis of TyG-BMI index with all-cause mortality. Restricted cubic spline analysis of univariate model of TyG-BMI index with 30-day **(A)**, 90-day **(B)**, 180-day **(C)**, and 365-day **(D)** all-cause mortality.

Based on RCS curve analysis, TyG-BMI index and 365-day all-cause mortality were found to have U-shaped relationships, with 320.1 being the inflection point, whereas the TyG-BMI index was not non-linearly associated with 30-day all-cause mortality (Figures 3A,D). Meanwhile, U-shaped relationships still existed between TyG-BMI index and 90-day and 180-day all-cause mortality, the inflection points were 311.1 and 316.5, respectively (Figures 3B,C). In addition, it could be seen that though there was a U-shaped relationship between TyG-BMI index and 90-, 180-, and 365-day all-cause mortality, the risk when TyG-BMI index was greater than the inflection point was much lower than the risk when TyG-BMI index was less than the inflection point.

### Subgroup analysis

**Figure 4:**
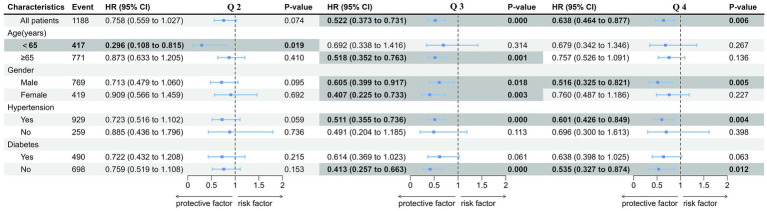
Forest plots of hazard ratios of TyG-BMI for the 365-day mortality in different subgroups. TyG-BMI index: Q 1 (TyG-BMI < 240.6), Q 2 (240.6 ≤ TyG-BMI < 278.8), Q 3 (278.8 ≤ TyG-BMI <329.5), and Q 4 (TyG-BMI ≥ 329.5). Forest plots of hazard ratios of univariate model in different subgroups including different age, gender, hypertension, and diabetes.

An investigation of the association between TyG-BMI index and 365-day all-cause mortality under different conditions was conducted, including age, gender, hypertension, and diabetes. The results showed that the risk for mortality (HR) in Q2 was significantly lower compared with Q1 only among patients less than 65 years old (and approached significance among those with hypertension), in Q2 risk was significantly lower only among patients more than 65 years old, those with hypertension, and those without diabetes, but both men and women, with no sex difference, while the risk in Q4 significantly lower only among men, those with hypertension, and those without diabetes. Risk in Q3 and Q4 also approached significance among those with diabetes (Figure 4). These results indicated that the predictive value of TyG-BMI index in 365-day all-cause mortality of patients with AMI was different among populations and needed to be further verified in different populations.

## Discussion

The retrospective study was the first investigation to examine the association between the level of TyG-BMI and all-cause mortality in critically ill patients with AMI. The results showed that TyG-BMI was significantly in relation to all-cause mortality in critically ill patients with AMI and found that the U-shaped relationships between TyG-BMI index and 90-, 180-, and 365-day all-cause mortality. These results indicated that both extremely low and high levels of TyG-BMI increased the risk of 90-, 180-, and 365-day all-cause mortality in patients with AMI, especially low level of TyG-BMI was associated with the greatest risk of all-cause mortality. These results may facilitate the development of clinical guidelines for reducing death among these patients.

Insulin resistance is the typical characteristic of metabolic syndrome and obesity. Rafael et al. (10) reported that IR seemed to significantly have an important short-term prognostic role in patients with AMI. A review suggested that the IR group had increased susceptibility to ischemia–reperfusion (I/R) injury. Inflammation, increase in oxidative stress, impairment of insulin signaling, and elevated basal oxidative stress may contribute to this process (19). The anti-inflammatory and antioxidant activities of insulin had been reported to have a beneficial effect on endothelial action and vascular wall function (20, 21). Insulin signaling has an important role in the regulation of blood glucose, and patients with IR are often accompanied by hyperglycemia. In our study, fasting glucose significantly increased in AMI patients with high TyG-BMI levels. IR may need to be synergistic with other clinical factors to increase susceptibility to AMI and worse the prognosis in patients with AMI.

It is well established that IR is strongly associated with the development of AMI. Meanwhile, the TyG index, a valid surrogate of IR, was assessed to significantly associated with the patients with AMI and could to be an independent risk factor for the occurrence and development of AMI (22). However, in our study, on the prognosis of all-cause mortality in critically ill patients with AMI after combining TyG index with BMI to form the TyG-BMI index, “U-shaped” relationships were found between TyG-BMI and 90-, 180-, and 365-day all-cause mortality in critically ill patients with AMI. Our explanation for these results were as follows, we could find that low TyG index was caused by low triglyceride or low glucose from the formula for calculating TyG index. It is possible that low fasting glucose is the cause of the association between low TyG index and all-cause mortality in individuals with AMI. The researchers found that low serum glucose (≤4.0 mmol/L) were associated with an increased risk of all-cause mortality, major cardiovascular events, and ischemic stroke in individuals without cardiovascular disease (23). In addition, some studies also indicated that low blood glucose levels were significantly associated with an increased risk of atrial fibrillation, diabetes, stroke, and major cardiovascular events (24–26). Studies also showed that patients with more than five episodes of hypoglycemia per year have a 61% higher risk of cardiovascular events compared to patients with fewer episodes of hypoglycemia, with the risk of cardiac arrhythmias, cerebrovascular accidents, and myocardial infarction increased by 65, 38, and 43%, respectively (27). Interestingly, it is reported that obesity increases the risk of cardiovascular disease (28). However, many studies also showed that the relationship between BMI and risk of disease was often described as “U” or “J”-shaped, and in some cases, those with a low BMI had a higher short-term risk of mortality than those who were overweight (29). This may be attributed to the fact that BMI was only calculated with height and weight, which did not provide a true assessment of adipose tissue distribution and body composition, and thus had some limitations in the evaluation of central obesity. Meanwhile, results of studies on the effects of low levels of lipids on cardiovascular disease were inconsistent. A prospective cohort study found that low level of low density lipoprotein cholesterol (LDL-C) below 70 mg/dL and low level of triglyceride was in relation to an increased risk of hemorrhagic stroke in women (30). Wu et al. (31) also found that low level of LDL-C would increase the risk of stroke. However, some studies have suggested that cardiovascular benefits continued to increase as LDL-C decreased even when LDL-C reached low levels. In our study, we found that the increased risk of all-cause mortality in patients with higher TyG-BMI index was much lower than that in patients with lower TyG-BMI index, suggesting that low level of glucose or triglyceride was associated with higher risk of 90-, 180-, and 365-day all-cause mortality in critically ill patients with AMI. Therefore, more clinical evidence is needed to validate our conclusions in the future. Meanwhile, increased BMI may attenuate the risk of all-cause mortality due to IR, and that low body weight increases the risk of all-cause mortality in critically ill patients with AMI. Interestingly, the “obesity paradox” has recently been reported in the literature regarding the effect of obesity on AMI patients, but there are no studies on patients with critically ill patients with AMI (32). Therefore, we believe that the “obesity paradox” may be a plausible explanation for our results in this study.

In our study, it seemed that the risk was lower in all subgroups and in all quartiles Q2-Q4, but the statistical significance was seen only for certain subgroups. The reduced number of subjects in certain subgroups possibly diminishes the statistical significance, but there could be also other explanations. We found that the risk with the lowest TyG-BMI was higher compared with those with the highest TyG-BMI only in non-diabetic individuals, hypertensive individuals and men, not in diabetic patients, non-hypertensive individuals and women. Several diseases are clinically associated with IR includes obesity, T2DM, metabolic syndrome, cardiovascular disease, and cancer (33), and IR is a typical and general characteristic of patients with T2DM. Therefore, the difference in prognosis by stratification of TyG-BMI index in the diabetic patients is not as significant as that in the non-diabetic individuals. It is reported that there is a synergistic effect of TyG index and hypertension on stroke, and a small proportion of the association between TyG index and stroke was mediated by hypertension (34). Meanwhile, hypertension is a great risk factor of all-cause mortality. Thus, our results may also reflected that there is a synergistic effect of TyG-BMI index and hypertension on all-cause mortality, and the risk of all-cause mortality due to different levels of TyG-BMI index may differ more significantly in patients with hypertension. IR is a phenomenon that can be found in both men and women and in particular, in the latter, it is found mainly after menopause. Premenopause, hormonal fluctuations during the menstrual cycle, and the presence of estrogen can affect insulin sensitivity (35). In our study, the mean age of the patients included was 73.03, so large fluctuations in sex hormones are particularly pronounced in women and may affect insulin sensitivity to a greater extent. Our findings support the idea that TyG-BMI, a good proxy for insulin resistance, may be weaker in predicting all-cause death in women than in men.

Neland et al. (36) proposed an interesting concept of the “threshold effect” of obesity in AMI. Overweight and obese patients had a large amount of adipose tissue accumulation, so they had sufficient energy reserves, which improved the prognosis of patients after AMI (36, 37). Our results also found that the “protective” effect of obesity was not observed in about 30 days, and gradually showed up as the extension of follow-up time. In addition, with the increase of BMI, it had a protective effect on patients with AMI within a certain level, but it turned into a harmful effect when it exceeded a certain range. Most likely, changes in cardiovascular risk burden, hemodynamics (increased total blood volume, increased cardiac output, and increased workload), and autonomic nervous system in obese patients outweigh the “protective effect” of energy reserves in adipose tissue in obese patients and increase the incidence of end events (36, 38, 39).

The mechanisms underlying the “obesity paradox” remain unclear. Firstly, adipose tissue accumulated in critically ill patients with AMI and was fully mobilized when necessary, where it could provide energy more efficiently than exogenous nutrients and could prevent muscle tissue depletion (40). Overweight and obese patients were often comorbid with hypertension and diabetes mellitus, and the dosage of medications needed to treat cardiovascular disease was easier to individualize and to develop a rational treatment plan in obese patients than in normal-weight patients (41, 42). In addition, for normal weight and low weight patients, antiplatelet drugs were often used at standard doses that were not adjusted for body weight, so there may be cases where excessive doses were used, which may lead to complications such as bleeding, which may also lead to higher risk of mortality.

Our study indicates that TyG-BMI, which combines TyG index and BMI, is an efficient clinical surrogate marker of critically ill patients with AMI. The management of critically ill patients in ICU is an important topic and also the focus of clinical work. As an easy parameter to obtain when patients enter ICU, TyG-BMI index can better prompt clinicians to identify high-risk patients in time, reduce mortality, and improve patient’s prognosis.

Several limitations still existed in our study. Firstly, the retrospective study was based on the observational data extracted from MIMIC-IV database; it was hard to clarify the causal relationship. Although a variety of variables were adjusted and subgroup analysis was conducted, we could not completely exclude the influence of potential confounders on the outcome. Secondly, the present study was a single-center study, which made it difficult to avoid kinds of bias. In view of the small sample size of the study population, more cohort studies with large samples were needed to validate our conclusions. Thirdly, the data of blood glucose and lipids were extracted as the first measurements of patients who admitted to ICU, it is not completely certain that the measurements were obtained from fasting patients. Fourthly, the MIMIC-IV database does not have the data about cause-specific mortality, so we only analyses the data about all-cause mortality of AMI patients. Our further studies would explore the association between TyG-BMI index and cause-specific mortality. Meanwhile, the study lacked the comparation of the effects of multiple IR resistance markers on critically ill patients with AMI.

## Conclusion

The present study found that the U-shaped relationship exists between the level of TyG-BMI index and the risk of all-cause mortality in critically ill patients with AMI, particularly associated with the 90-, 180-, and 365-day mortality, but not statistically significantly associated with the 30-day all-cause mortality; even it followed the similar pattern. Further studies with cause-specific mortality and involving specific subgroups of patients are needed to validate and better clarify our results.

## Data availability statement

Publicly available datasets were analyzed in this study. This data can be found at: https://mimic.mit.edu/.

## Ethics statement

The requirement of ethical approval was waived by Institutional Review Board at the Beth Israel Deaconess Medical Center for the studies involving humans because the collection of patient information and creation of the research resource was reviewed by the Institutional Review Board at the Beth Israel Deaconess Medical Center, who granted a waiver of informed consent and approved the data sharing initiative. And the ethics could be found in https://physionet.org/content/mimiciv/2.2/. The studies were conducted in accordance with the local legislation and institutional requirements. The ethics committee/institutional review board also waived the requirement of written informed consent for participation from the participants or the participants’ legal guardians/next of kin because the collection of patient information and creation of the research resource was reviewed by the Institutional Review Board at the Beth Israel Deaconess Medical Center, who granted a waiver of informed consent and approved the data sharing initiative. And the ethics could be found in https://physionet.org/content/mimiciv/2.2/.

## Author contributions

CL: Writing – review & editing, Writing – original draft, Visualization, Software. QL: Writing – review & editing, Software, Methodology, Investigation, Formal analysis, Data curation. ZW: Writing – review & editing, Formal analysis, Software. SD: Writing – review & editing, Formal analysis, Data curation. QM: Writing – review & editing, Supervision, Investigation.
